# Leukemia and Heart Disease: Lessons at the Intersection of the Most Feared CHIP Outcomes∗

**DOI:** 10.1016/j.jaccao.2022.01.094

**Published:** 2022-03-15

**Authors:** Philipp J. Rauch, Benjamin L. Ebert

**Affiliations:** a Department of Medical Oncology, Dana-Farber Cancer Institute, Boston, Massachusetts, USA; b Broad Institute of Harvard and MIT, Cambridge, Massachusetts, USA; c Howard Hughes Medical Institute, Boston, Massachusetts, USA

**Keywords:** acute myeloid leukemia, aging, clonal hematopoiesis, coronary artery disease, inflammation

**Figure:**
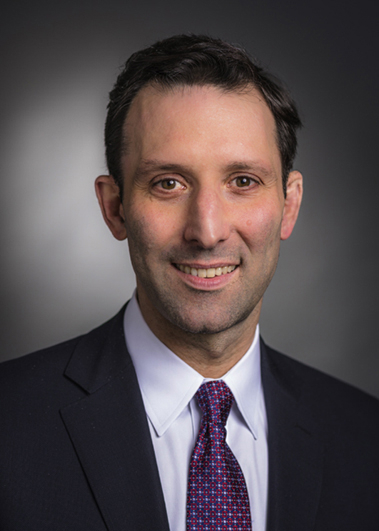

Mutant blood cells can be associated with both malignant and nonmalignant outcomes. Clonal hematopoiesis, long known to occur in the context of hematologic neoplasia,^1^ describes an outsized contribution of a clone derived from a single hematopoietic stem cell and was more recently found to be very common and strongly age-associated in individuals without a known blood cancer diagnosis.^2^ When the clone’s competitive advantage is driven by a mutation in a gene recurrently mutated in hematologic malignancies and the variant allele fraction (VAF), a measure of the clone’s contribution to the peripheral leukocyte pool, exceeds 2%, this phenomenon has been termed clonal hematopoiesis of indeterminate potential (CHIP).^3^ Importantly, the presence of CHIP is associated with an increased risk of adverse outcomes^2^^,^^4^^,^^5^: the condition was found to confer a substantially increased risk of subsequently developing a hematologic, most commonly myeloid malignancy, the most feared of which is acute myeloid leukemia (AML).^6^ A striking additional finding was an increased risk of cardiovascular disease among CHIP carriers, likely driven by enhanced inflammation in terminally differentiated blood cells and thought to be causally related to the presence of the mutated clone.^4^^,^^7^

In this issue of *JACC: CardioOncology*, Calvillo-Argüelles et al^8^ ask how the presence of CHIP-related mutations at the time of a diagnosis of AML impacts the frequency of subsequent cardiovascular events (CVE) in patients. Hypothesizing a connection is logical because AML can arise as the culmination of CHIP, and by definition is associated with a dramatic expansion of leukocytes carrying the culprit mutation. To address this question, the investigators elegantly exploit the fact that AML is caused by specific mutations, some of which occur in CHIP and some of which do not.^9^ This enables them to divide patients with AML into 2 groups, 1 with CHIP-related mutations, and 1 without. After adjusting for covariates, they then compare the frequency of CVEs in the 2 groups in a retrospective cohort design, finding that such events were more common in patients with CHIP-related mutations who were considered eligible for intensive chemotherapy, but not in the whole cohort. They also found that incident CVEs after AML diagnosis were associated with an increase in all-cause mortality.

This study is important in several different ways: for one, it is biologically informative. The fact that patients with AML and a CHIP-related mutation have higher rates of CVEs than those with other mutations lends further support to the notion of a causal relationship between mutation and cardiovascular disease. Additionally, prior studies have demonstrated that the risk of adverse outcomes in the context of CHIP is related to allele burden, with the majority of risk being confined to individuals with a VAF of >10%.^4^ Because the size of the malignant clone, and therefore the VAF of the mutated allele, in patients who have progressed to AML will often be very high, it is plausible that patients with CHIP-derived AML would be at particular risk. And lastly, a recent study has demonstrated that diseases of “inflammaging”^10^ often precede a diagnosis of myeloid malignancy.^11^ This is consistent with CHIP mutations being associated with inflammation even in the context of AML, leading to the increased risk of cardiovascular disease reported here.

A prima facie surprising aspect of the study is the fact that a difference in incident CVE is detectable in the comparatively short period of time after diagnosis of AML. However, there are several plausible explanations for why this may be occurring, all ultimately related to the inflammation hypothesis of cardiovascular disease.^12^ First, at the time of diagnosis with AML, patients have likely had clonal hematopoiesis for many years, allowing for deleterious effects, such as development of atherosclerotic plaque, to manifest. Second, AML has been shown to be associated with an inflammatory state,^13^ which may act as a catalyst precipitating acute events. Furthermore, some AML therapies, most notably the anthracyclines, cause cardiotoxicity.^14^ Third, despite the chronic nature of the underlying processes in atherosclerotic plaque development, its evolution is not necessarily a gradual process. Longitudinal studies in humans undergoing 4 coronary angiograms per year demonstrated that in many cases, progression occurs in sporadic surges of growth,^15^ which could explain the pathology observed here.

From a clinical perspective, the presented findings may help hematologists and oncologists taking care of patients with AML to evaluate the risk of a CVE in patients undergoing treatment. Recognition of risk might justify increased monitoring and/or intensified management of pre-existing risk factors such as hypertension or dyslipidemia. Such measures should be tested in a prospective and ideally randomized setting before becoming standard practice, as risks are conceivable in these medically complex patients even with relatively “benign” interventions.

Like many interesting studies, this paper raises as many questions as it answers.^8^ CHIP has recently been associated with inflammatory pathologies outside of the cardiovascular system, including chronic obstructive pulmonary disease,^16^ and it would be interesting to determine whether patients with AML and CHIP-related mutations also experience more frequent acute pulmonary events. Somewhat surprisingly, at the level of individual genes, the investigators report the strongest effects on CVE for mutations in *ASXL1* and *TP53*, whereas mutations in *TET2* and *DNMT3A*, robustly associated with increased cardiovascular disease in other studies, show minimal to no increase in hazard ratio. Although this may be part of the underlying biology, it could also point to partially uncontrolled confounding that can never be fully accounted for in a retrospective study. In particular, mutations in *TP53* may reflect prior exposure to cytotoxic therapy, which could itself predispose to cardiovascular disease.^17^ Overall, both further mechanistic exploration and prospective validation of the observed effects are warranted, and hold promise to inform and refine the management of cardiovascular risk in patients with AML.

## Funding Support and Author Disclosures

Dr Ebert has received research funding from Celgene, Deerfield, Novartis, and Calico; has received consulting fees from GRAIL; and is a member of the scientific advisory board and a shareholder for Neomorph Therapeutics, TenSixteen Bio, Skyhawk Therapeutics, and Exo Therapeutics. Dr Rauch has reported that he has no relationships relevant to the contents of this paper to disclose.
